# The Hospital. Nursing Mirror

**Published:** 1898-07-23

**Authors:** 


### "The Hospital" Nursing Mirror.

Being the Nursing Section of "The Hospital."

[Contributions for this Section of "The Hospital" should be addressed to the Editor, The Hospital, 28 & 29, Southampton Street, Strand, London, W.C., and should have the word "Nursing" plainly written in left-hand top corner of the envelope.]

### News from the Nursing World.

### NEW NURSING QUARTERS AT QUEEN CHARLOTTE'S HOSPITAL.

The new structural additions to Queen Charlotte's Hospital are immense improvements. In addition to the new nurses' home just opposite the hospital, the foundation-stone of which was laid a fortnight ago, the topmost storey has been raised a couple of feet and another one built above it. The floor which of old afforded accommodation to the sisters now provides five excellent wards for the reception of patients; it gives the sisters a sitting-room; the nurses a cloak-room, with pegs, lockers, and lavatories; the matron a linen-room, and the whole floor a ward kitchen. The last-named is an acquisition all the more appreciated because none of the other floors possess one. On the same floor, but shut off by doors, are the servants' sleeping apartments, with bath and lavatory for their use. The new floor gives the sisters handsome bed-sitting-rooms, in the adornment of which they display a good-natured rivalry. The windows are ingeniously contrived: the centre casement opens in four fanlights, which can be opened and closed independently the one of the other. The side-lights open towards the fans, and so cut off any draught that may be felt. The walls are painted and varnished, the dados being chocolate, the upper walls in the wards white, and those in the corridors and offices cream-coloured. The sisters' rooms are prettily tinted. The next improvement is to be a lift, which will be put in as soon as possible. All the fittings are of the latest sanitary fashion.

### NURSES AT THE GREAT NORTHERN CENTRAL HOSPITAL.

The nurses took a graceful part in the ceremony of the 19th inst., when the circular ward of the Great Northern Central Hospital was opened for use. They formed an effective choir, singing the hymns with sweetness and power. After the ceremony they waited on the guests in the refreshment rooms. The new ward involves the employment of five more nurses, and the matron has fortunately succeeded in securing them, and in making preparations for the training of extra probationers. The patients will all be in possession of the ward now, and on Sunday the vicar will hold his first regular service in it. He made known the want of a harmonium for use at these services, and before he left the ward one was offered for the purpose. The ward is a pretty one; the lockers of light oak, and the brackets over the head of the beds are excellent. The quilts are of linen, with broad alternate stripes of red and white, with the initials of the hospital interwoven. There is room for plants on the window ledges, and the white marguerites and red geraniums on them produced a pleasing effect.

### "THE HOSPITAL" CONVALESCENT FUND.

Contributors to the Convalescent Fund will be glad to hear that their offerings have enabled two more nurses to obtain a much-needed change. We print extracts from their letters in "Everybody's Opinion," and take this opportunity of thanking the principals of the homes to which they were sent for their kindness to them. We should also like to draw our readers' attention to the fact that our balance has reached a lower ebb than we like. Each nurse sent to a home costs about 35s.—a very small sum for so great a boon—but unless there is this sum in our exchequer for each applicant, we cannot entertain new cases. Want of generosity is not a characteristic of the generality of nurses, and we have no doubt that a mere mention of this difficulty will lead to our coffers being refilled.

### ANOTHER BENEVOLENT AGENCY.

Last year the Distressed Gentlefolks' Aid Association was founded, with the object of carrying succour to a class most difficult to reach. H.R.H. Princess Christian is the patron, and the names of the Bishop of London and Mrs. Creighton stand at the head of an influential list of councillors. During the past twelve months—from May 5th, 1897, to May 5th, 1898—subscriptions and donations amounting to £760 have been received, and £42 have been returned by persons who gratefully acknowledge their indebtedness to the society. The two essential qualifications are that the applicants must be "gentlefolk" and "distressed." There is no system of election by vote, the only priority of claim being accorded to age and infirmity. Grants have been made to 105, and weekly allowances to 36 cases. The recipients of this bounty are asked to regard the help thus given as a loan, which may be refunded when brighter days come; and the prompt repayment in several instances has been most cheering. Sometimes a friend or relative meets the weekly grant with an equal amount, and thus ensures the necessities of life to a distressed gentleman or woman. The work appeals strongly to the sympathies of those who know the martyrdom expressed under the term "distressed gentlefolk." Full particulars of the society may be obtained from the hon. secretary, Miss Finn, 75, Brook Green, London, who will also gladly receive contributions either of money or clothing. The Princess of Wales and the Princess Victoria of Wales have sent donations and several garments made by the latter for distribution.

### SUFFOLK NURSES HOLIDAY-MAKING.

By the kindness of Lady Constance Barne, thirteen of the fourteen nurses on the staff of the Suffolk Nursing Association recently enjoyed a delightful holiday at Gray Friars, Dunwich. After dinner some explored the ruins of the monastery and church, some enjoyed the sea breezes from the cliff, and some went for a sail. After tea Archdeacon Gibson gave a sympathetic and encouraging address, and Lady Constance presented each nurse with a medal to be worn as long as she remained a member of the association. The guests then took part in croquet and other out-door games, and Lady Constance photographed them. An entertainment was given after supper by some ladies, and the nurses left the next morning for their various duties. Nearly all the nurses had received hospital and district training through the scholarships granted by the West Suffolk County Councils. Other nurses are now being trained by the same means, and districts are awaiting their services.

### HANLEY NURSING ASSOCIATION.

At the annual meeting of this association, the Duchess of Sutherland was re-elected president, and the other officers were reappointed. During the year the society has been affiliated with Queen Victoria's Jubilee Institute for Nurses, and £339 have been collected as a memorial of the Diamond Jubilee. The sum is not enough for the purpose desired, namely, the purchase of a house as a nurses' home, and the committee therefore ask for further subscriptions. The work of the nurses has largely increased, and signs are not wanting that the townsfolk are interested in it. Five hundred cases were attended.

### RED CROSS NURSES FOR TAMPA.

The Red Cross Society for the Maintenance of Trained Nurses has commissioned the Red Cross Relief Committee to transport fifty-seven trained nurses to Tampa, and maintain them for as long a time as may be needed. Miss Barton is already there, prepared to go to the front at a moment's notice. Only trained nurses giving their services were accepted. Miss Maud Cromelieu received and registered the nurses volunteering for the work at the hospital in West 100th Street, New York. The "Maintenance Auxiliary" commissioned Mrs. F. K. Sturgis to pay the treasurer of the Relief Committee 2,205 dols., the expenses for one month in advance.

### A NOVEL IDEA.

A novel idea, with the excellent result of putting £25 into the coffers of the National Society for Prevention of Cruelty to Children, was recently put into practise at Mitcham. A lady possessing a charming garden organised a "cap and apron sale," providing tea at the moderate cost of sixpence a head. A few friends sang some songs and glees. Numbers of those present had cycled out from town.

### OPENING THE NURSING HOME AT ASCOT.

The Duchess of Albany opened the Royal Victoria Cottage Nursing Home on the 12th inst. She was attended by Miss Heron-Maxwell and Sir Robert Hawthorn Collins. Mrs. Liddell, to whose exertions much of the success of the enterprise is due, received her. The site was presented by the late Dean Liddell and General Crutchley, both of whom have died within the last six months. The home contains three wards of two beds each, two dormitories, a nurses' sitting-room, several single bed-rooms, an operating theatre, and an isolation-room, which has only an out-of-door communication by means of a separate staircase. All the rooms are carefully ventilated and neatly furnished. The Duchess was presented with a bouquet and with a key bearing the same design as the foundation-stone, namely, a monogram, V.R.I., with the rose, shamrock, and thistle, surmounted by a crown. The date upon it was that of the laying of the stone, November 24th, 1897. Miss MacDowal has been appointed matron, and is assisted by a fully trained surgical nurse and an efficient staff.

### NURSES' LIBRARIES.

Exclusion from public libraries is a hardship that often falls to the lot of a nurse, but one which does not always press on public attention as it ought to do, because it is not unfrequently ignored. Everyone sees how impossible it is for one whose calling is the care of those afflicted with the various ills that flesh is heir to, to guarantee that between the date of her taking out a book to that when it is returned, the volume shall never be exposed to the chance of infection. It is equally obvious, even to an optimistic socialist, that selfish individuals will carelessly sacrifice the health of the community to a few hours' pleasure; therefore, the community must, where it can, take thought for itself. The action of the Lewisham Public Libraries Committee, in setting aside 200 volumes for the use of the Infirmary nursing staff upon the guarantee of safe custody given by Canon Rhodes Bristow, is, therefore, a prudent one, and we observe with satisfaction that upon Canon Bristow's retirement from the office of Chairman of the Board, the Clerk was directed to give the required guarantee for the safe custody of the books on behalf of the Board. The nurses by this means are admitted to the privileges enjoyed by the rest of the community, and the community is assured that the public health is, as far as possible, safeguarded.

### A NEW NURSING SOCIETY.

Preliminary steps to the formation of a district nursing association for the districts of Camberwell and Walworth have been taken by Lieut.-Colonel Dalbiac, M.P., and Mrs. Dalbiac, and by Mr. James Bailey, M.P., and Mrs. Bailey. The sum of £250 is necessary as a commencement. It is proposed to begin work with four nurses.

### SHEFFIELD: A NURSING REPORT.

Mr. S. G. Richardson presided over the annual meeting of the supporters of the Sheffield Nurses Home and Training Institution. The nurses' earnings for the year amounted to £1,531, a slightly smaller sum than that of the previous year, owing to the illness of some of the staff. The earnings of 117 weeks were lost from this cause. The work of the district nurses is greatly appreciated by the poor, and an appeal was made to the public for additional contributions.

### SHORT ITEMS.

Princess Henry of Battenberg has signified her intention of attending a meeting in connection with the Victoria Nursing Society. Her Royal Highness is the president.—The Duchess of Albany opened the Royal Victoria Nursing Home at Ascot last Tuesday.—A bed has been set apart for a soldier, or for a soldier's wife, widow, or child, at the Somerset County Hospital for a period of five years, and an appeal is now being made to raise about £800 to endow this in perpetuity.—The offer of the Essex County Nursing Association to supply a nurse on favourable terms to Grays has been accepted, and arrangements have been made for one to begin work at once.—Since the School Nurse began to visit Vere Street Board School on Friday mornings, cases of ophthalmia have decreased in number in a satisfactory way; the teachers kindly applying the eye lotion regularly.—Miss R. M. Saward, of the Plymouth Workhouse Infirmary, has taken the L.O.S. certificate.—A jumble sale realising £25 in support of the trained nurse as Clophill, was recently held in the rectory grounds.—The Woolwich Board of Guardians have sent their usual subscription of £25 to the Woolwich, Plumstead, and Charlton Nursing Association.—The bazaar in aid of the Littlestone-on-Sea Convalescent Home realised about £800.

### Notes on Gynæcological Nursing.

By ARTHUR E. GILES, M.D., B.Sc., F.R.C.S., Assistant Surgeon Chelsea Hospital for Women.

### II.—SEPSIS AND ASEPSIS CONTRASTED.

I SHALL, perhaps, best impress the nurse with the facts dwelt upon in the last lecture by drawing pictures of two modes of carrying out an operation, by way of contrast.

1. A SEPTIC OPERATION.—In a room not over clean, with a carpet that has forgotten its last beating, the patient is placed on the operating table. She has had her hands and face washed, but nothing more. The operation is "ovariotomy." The surgeon and his assistant wash their hands, using a small nail-brush that does duty for the family, after which they proceed to arrange the patient and move the table nearer the light. While the anæsthetist is getting the patient "under," the surgeon thoughtfully pulls his beard; the assistant ties up a boot-lace which has got loose. The nurse meanwhile arranges on a tray the instruments and sponges, which have been brought loose in the surgeon's bag. On some of the sponges and in the joints of the instruments specks of blood can be seen, from an operation on the previous day. After these things are arranged the nurse rinses her hands, and helps the surgeon on with an old operation coat which he carries with him to save splashing his ordinary clothes. The patient's abdomen is exposed and towels are placed across the body above and below. The patient shows signs of vomiting; the surgeon places the knife, which he had already picked up, in his mouth, while he helps to roll the patient over on one side. The assistant empties a bowl which had some flowers in it and hands it to the anæsthetist. One of the towels placed on the patient falls off during these proceedings; it is picked up and rearranged. The operation begins. Soon there is a knock at the door, and the nurse, who is looking after the sponges, goes and opens it, and takes in a hot-water bottle, and then resumes her work without washing her hands. The surgeon drops a forceps on to the floor; his assistant picks it up and seizes a bleeding vessel with it. Presently the surgeon asks for a silk ligature on a needle; the silk frays out a little, and the nurse being pressed for time bites off the end and threads it. There is, perhaps, some unusual condition in the abdomen, and the surgeon asks a visitor who is present to put his hand in and feel; the visitor has not washed, and as he puts his hand in, his coat sleeve touches the wound in the skin. Presently some water is wanted for flushing out; that in the kettle is too hot, so cold is drawn from the tap and added. At the close of the operation gauze is wanted for the dressing; it had not been put out, so the nurse opens the surgeon's bag, gets out the gauze which was lying loose in it, and hands it to the assistant, who places it on the wound. Finally the patient is got back to bed. The next day she appears collapsed; in the evening she has a rigor. Next morning her temperature is 103, she has a thready pulse, a brown, dry tongue; her abdomen is distended like a drum and very tender. In the evening sepsis has done its work and the patient quietly breathes her last, whilst all concerned grieve and wonder why so straightforward and simple an operation should have proved so rapidly fatal. Was it shock? Did she lack stamina? No; but she was infected, poisoned a hundred times over during the operation, and the miracle would have been her survival.

2. AN ASEPTIC OPERATION. — The room has been thoroughly cleaned and scrubbed, and disinfected by means of an Alformant lamp. The sheet and mackintoshes on the operation table have been disinfected. The patient has had a warm bath, after which the abdomen has been washed with soap and a nail brush; after the pubes has been shaved another wash is given with soap, then with corrosive sublimate, 1 in 1,000; a compress of lint wrung out of the same solution is then placed on the abdomen and kept in place with protective and a sterilised many-tail bandage. The instruments and dressings are placed in the steriliser. The nurse, who wears a linen overall with short sleeves, takes off her rings and scrubs her arms, hands, and nails for ten minutes; she then soaks and scrubs them in a sublimate solution, 1 in 1,000. She then lifts out the instruments from the steriliser, and with forceps places them one by one in a clean tray containing boiled water. The dressings are transferred to a sterilised towel and wrapped up ready for use. The surgeon and his assistants, having taken the precaution of a morning bath, put on sterilised linen jacket and apron or overalls, and prepare their hands. Meanwhile the patient is brought in; the abdomen is exposed; sterilised towels are wrung out of carbolic acid, 1 in 40, spread on mackintoshes, and placed above and below the place of operation, while dry sterilised towels are tucked in at the sides. Henceforth neither the operators nor the nurses touch anything that has not been disinfected. The compress is removed from the abdomen, and the surgeon begins. A gauze sponge is asked for; these are kept, ready sterilised, in a glass jar. The nurse removes the lid of the jar by holding it with a sterilised pad of gauze prepared for such a purpose. Coming back, her hand accidentally touches the coat of a visitor; she proceeds at once to disinfect her hands again. The surgeon and his assistant rinse their hands at frequent intervals in sterilised water, renewed each time; instruments are not allowed to remain lying about on the towels, blood-stained, but are frequently washed and replaced in the sterilised water. Perhaps a forceps slips and falls on the floor; it is allowed to remain there, or some person not concerned in the operation picks it up and places it apart. The operation is thus brought to a close with the minutest and strictest attention throughout to all the requirements of aseptic surgery. The dressings are taken out from their towel and applied; a sterilised many-tail bandage keeps them in place, and the patient is put back to bed. The next few days are uneventful; a slight rise of temperature on the day following operation marks the reaction from shock, after which the temperature remains normal. The patient is comfortable and cheerful, there is no abdominal distension, no vomiting, and the pulse is good. On the eighth day the dressings are removed for the first time; the wound is found dry and firmly healed, and the surgeon removes the stitches. A week later the patient is up, and at the end of the third week goes home.

In this way we may contrast the operation and its results when asepsis is maintained with the opposite condition of things On every score the argument is strongly in favour of the former. Even when the want of care is not followed by a fatal result, convalescence is likely to be stormy and interrupted, whilst any suppuration in connection with the wound may delay recovery for weeks and even months.

A word to the wise sufficeth.

### Presentations.

MISS MORANT, for the last nine years Lady Superintendent of the National Sanatorium, Bournemouth, on the occasion of her marriage, was presented by the committee with a cheque for twenty-five guineas. The chaplain gave her a carved rosewood secretaire; the resident medical officer and nurses a brass gong and card tray; the female patients a plated inkstand; the male patients, pictures; the porter and his wife, a hand-painted fire screen; and the servants, a plated butter dish. Miss Morant takes with her the sincerest wishes for her happiness.

### Nursing in Diseases of the Throat, Nose, and Ear.

By MACLEOD YEARSLEY, F.R.C.S.Eng., Assistant Surgeon to the Royal Ear Hospital, Surgeon-in-Charge of the Department for Diseases of the Throat, Nose, and Ear, the Farringdon General Dispensary, Hon. Surgeon for Diseases of the Throat and Ear, the Governesses' Home, &c.

### I.—INTRODUCTION.

IN the present day, when our knowledge of medicine and surgery has so increased as to render specialism a necessity and each system in the body has a vast literature of its own, the nurse must keep pace with the surgeon if the patient is to be adequately treated, and some knowledge of each speciality should therefore be included in her training if she wishes to be in a position to undertake any case at a moment's notice. If it be an eye operation, she should know something of ophthalmic nursing; if it be a mastoid case, she should be acquainted with the methods of the otologist; and when a nurse has to look after the instruments needed for an operation she will find herself embarrassed if she has no knowledge of those particularly required for the operations by the specialist.

Just as no man can become a good specialist unless he has acquired a sound knowledge of general medicine and surgery, so is it impossible for the nurse to make herself efficient in the care of special cases unless she has been in the first instance well trained in the general principles of nursing. The management of mental cases, the care of fevers, of patients who have undergone abdominal operations, or of gynæcological cases have for some time past been recognized as branches of nursing which require special knowledge and aptitude, and the same may now be said of ophthalmic nursing. Unless, however, a nurse has received some part of her training in a hospital devoted to diseases of the throat, nose, and ear, her knowledge of the management of such patients, except perhaps of tracheotomy cases, is small indeed, for, just as otology, laryngology, and rhinology are neglected in the curriculum of the student, so are they disregarded in the training of the nurse.

The object of these papers, therefore, is to endeavour to remedy, at least in part, this defect in the instruction of nurses, and if they arouse a greater interest in the care of throat, nose, and ear cases, they will not have been written in vain. The nurse who wishes to gain a further insight into the subject is strongly advised to take out a course of training at some special hospital, where she can obtain practical instruction, for theory, although a valuable aid to practice, is worth little without it.

It is taken for granted that the reader has already received training in elementary anatomy and physiology and the principles of nursing, and it would, therefore, be beyond the province of these articles to enter into those matters which are general to nursing. The making of beds, keeping of notes, temperature charts, &c., will not therefore be touched upon, except in their particular relations to the special subject under consideration. It is in such matters as the application of medicaments to the ear or nose that the nurse is usually so lacking in knowledge; even the simple operation of syringing is one that she seldom performs efficiently. The writer, when in charge of the ear department of a general hospital, was usually given a different nurse each week, and he was surprised to find that not one of them was able to syringe an ear properly, and as each one had consequently to be specially instructed, the help they afforded was hardly commensurate with the time spent in teaching them, especially as the number of patients to be dealt with single-handed was large, and often necessitated four hours' continuous work.

The reason for this lack of training in this special branch is easy to find. In many general hospitals there appears to be a curious distrust or dislike of specialists in these diseases, and an idea prevails that "anyone can do ears." This has led to placing the ear department in the charge of a junior assistant surgeon—a policy which has largely contributed to the formation of special hospitals. These special institut.ons, for whose existence the short-sighted action of the general hospitals is largely to blame, monopolise an enormous number of cases, which, if adequately treated at the latter charities, would have been invaluable as teaching material. As it is, a surgeon whose experience of the special department placed suddenly in his charge is practically *nil* can hardly be in a position to teach the subject either to students or to nurses.

### Nursing Arrangements at the Victoria Park Hospital.

THE whole system of nursing at the City of London Hospital for Diseases of the Chest at Victoria Park, E., has been reorganised. The nursing staff will number thirty, including four sisters, two charge nurses, twelve staff nurses, and twelve probationers. A sister, assisted by a charge nurse, five staff nurses, and six probationers will take care of the men's corridor, and a similar staff of the women's corridor; the third sister will be night superintendent and the fourth will act as assistant matron and home sister. The salaries range as follows: Probationers, for the first year, £10, for the second, £16; staff nurses, £25—27; sisters from £36—45. Probationers not under 22 years of age will be received for two years' training. The matron then helps them, if she can, to obtain a third year's course of instruction in a general hospital, at the expiration of which the committee of the Victoria Park Hospital, upon receiving a recommendation from the matron under whom this third year has been spent, will grant a three years' certificate. The Secretary of the Royal British Nurses' Association has informed the matron of the Victoria Park Hospital that this certificate will be reckoned sufficient to admit the holder to registration and membership of the association. The year's course of instruction or longer in a general hospital or infirmary may be taken previous to entering the Victoria Park Hospital as probationer, but the testimony of the matron under whom it was taken, and the two years' course at the Victoria Park Hospital itself, are essential to obtaining the certificate. The prospects of promotion to the charge nurses are that in due course they may become night sister and then home sister, when they will receive instruction in the duties of matron, and undertake the matron's work in her absence. The course of training comprises lectures by a member of the visiting medical staff, clinical teaching by the resident medical officer, and practical instruction by the matron. The domestic arrangements for the comfort of the nurses are to be improved. Each nurse has a separate bedroom, and a large handsome room on the ground floor, shut off from the rest of the hospital, is now in the hands of the painters, preparatory to being converted into a sitting-room. In September there will be vacancies for probationers and staff nurses. These new regulations will certainly fill a need in the nursing world, as many probationers with a year's training will be able to obtain the three year qualification which is now essential to a nurse's success.

### Wants and Workers.

[THE attention of correspondents is directed to the fact that "Helps in Sickness and to Health" (Scientific Press, 28 & 29, Southampton Street, Strand, London, W.C.) will enable them promptly to find the most suitable accommodation for difficult or special cases.]

*L. H.*—The Union Infirmary, West Hartlepool, would be glad to buy a model of the pelvis for teaching purposes. It must be in good condition and cheap.

### Work and Workers in India.

By Nina.

I have been asked—as having had a little experience in this way—to say something of nursing work and life in India, which offers a wide field for nurses, the area of which, in view of recent plague and frontier troubles, is not likely to be diminished. Under these circumstances I propose, metaphorically, to take the nurse from England to the scene of her labours, helping her with brief but practical hints as to uniform and general equipment. Most of these remarks will apply equally well to any tropical climate, and as such may not prove unacceptable to the general reader.

The private nurse, especially if well versed in obstetrics, need never lack cases, and there is room for more than one nursing institution where young married ladies could be received for the trying ordeal that often has to be undergone in an Indian hotel, which at its best differs widely from the average English one. Instruments such as are contained in the nurse's wallet should all be carefully chosen with a view to their heat and damp-resisting powers, only nickel plated tools will withstand the inroads of rust. A hypodermic syringe and plentiful supply of needles will prove a boon, as in addition to the trouble of getting a special syringe fitted there is often untold delay. Those made of vulcanite warp. Jaconet as we know it at home is invaluable, and should have a place in every nursing kit.

It matters little which branch of nursing is to be undertaken, whether civil, military, plague, or private, the primary qualification is the same, namely, professional ability combined with the highest moral and physical calibre.

These points, although equally necessary at home, bear a double significance abroad, where moral obliquity is thought to be excused by the plea of the enervating climate and where frequent supervision is ofttimes practically an impossibility. Truly, it is sometimes difficult to be consistently conscientious with the thermometer registering 100 or 110 degrees Fahrenheit in the shade. To come to our third point, physical fitness. If the nurse receives her appointment in England she will have been examined as to this, but if not, will she have given the matter due consideration?

There is no need to recruit the army of the sick from our side of the water, and a nurse "upon her own account," unless in assured possession of the necessary sum at her bankers, might find the return journey a serious matter. In a land where the animal food is killed and cooked within a few hours digestion should claim the candidate's first attention, as much time, expense, and possibly some pain will be averted by dental repairs being made prior to departure. I can speak feelingly upon this subject, having had occasion while there to get an exposed nerve killed. The "best dentist's" methods proved but half effectual, and the nerve was finally taken away in twelve pieces. I do not say that the man was clumsy or that his instruments were prehistoric, but I can say the process was painful and the charge high!

We will now turn to the subject of trunks, which should be asked for of "regulation size," a passport to their admission into one's cabin, where the necessary outfit for a three weeks' voyage should be kept. Heavy baggage descends to the "hold," and is lost sight of until one lands, while moderately large packages are stored in the baggage room, to which access is allowed once or several times a week according to the line travelled upon. Strong tin or tin-lined trunks are the best; they resist alike the ravages of the white ant and blue mould. All superfluous straps and padlocks should be avoided, and the name painted in full upon the lid.

A large hold-all, a cushion, a travelling rug, an umbrella—with loose white cover, and a deck chair are indispensable, adding much to one's independence and comfort. Better than travelling cap or hood is the black lace scarf or woollen fascinator, which, being pinned or tied closely to the head, defies the efforts of the wind to disarrange one's hair, and renders the after-dinner moon-lit seat "in the bows" a time of calm enjoyment.

In a place where after all there is a good deal of enforced monotony, dress holds an important position, the evening meal—unless in the case of second-class passengers—necessitating regulation toilet. This is easily varied by some smart evening blouses, which answer well for the concerts, &c., given on board. Old costumes should not be despised, and will find their value often in the early days of the voyage, while all warm clothing, in addition to that required for the welcome trips on shore, should be ready to hand. A spare corner in the cabin trunk should be filled with a box of one's favourite biscuits or sweets, and the handbag should contain eau-de-cologne, smelling salts, antipyrin or phenacetin tabloids, and some stimulant to meet emergencies. A white sailor hat of Panama or other light straw is the best, and should be accompanied by a stock of ready-made bows to match the ties and waistbelts intended to be worn.

Uniform should be made of light cotton, and if trimmed with any other colour this should be detachable. With such uninterrupted stretches of fine weather white dresses are most useful, and with a coloured skirt will serve for mufti. The brightest colours, which, mixed with our sombre English hues would appear crude, can there be worn with impunity, by people, too, of uncertain age! Quantity is not a matter for deep consideration, as washing in most regions is done for the nominal sum of five or six rupees (7s. or 8s.) per month, the number of articles sent often being twenty-four to thirty-six a week. The writer found eight dresses and eight caps an average number, even in plague work, which is less clean than the other branches. Linen-faced cotton, or cotton itself, lasts much longer than linen, and a plentiful supply of aprons should be made of this. Blue, contrary to home experience, washes much better than pink, which in the strong light fades very rapidly.

Acquaintanceship on board soon develops into friendship, and autograph and birthday books will be in requisition before the completion of the voyage.

### Appointments.

### MATRONS.

Children's Hospital, Athens.—The Crown Princess of Greece (Duchess of Sparta) has made the following appointments in connection with the Children's Hospital which has lately been opened in Athens: As Lady Superintendent, Miss Maud Calvert, matron of the Branch Seamen's Hospital in the Royal Victoria and Albert Docks, E.; Sister, Miss Catherine Russell Fryer, charge nurse of the same institution. Miss Calvert was trained at Guy's Hospital, and formerly matron at Newark Hospital. The appointments date from November 1st next.

Miss C. Glover, the newly-appointed Matron of Kaiser-el-Aini Hospital, Cairo, kindly gives us the following particulars of her training. She was staff nurse in the Leopold ward at St. Thomas' Hospital, and her duties as home sister at the Hospital for Sick Children, at Great Ormond Street, and at the Canterbury and Kent Hospital were temporary ones for short periods only—the one for five weeks and the other for two months respectively.

Royal Eye Hospital, St. George's Circus, Southwark, S.E.—Miss Kate Elizabeth Norman has been appointed Sister-Matron to the above hospital. She was trained and afterwards for two years on the staff of the London Hospital. She has since held the post of sister at the Royal London Ophthalmic Hospital, Moorfields, E.C., for two years.

A Correction.—Weston-super-Mare Hospital.—Miss Sherring's appointment was erroneously stated to have been to the Swansea Hospital in our last issue. She was appointed, on July 4th, Matron of the Weston-super-Mare Hospital from the Swansea Hospital.

### A Book and its Story.

### HELBECK OF BANNISDALE.

From a weird and shadowy background of marshland and fell, in the gathering twilight of a stormy March day, Helbeck of Bannisdale,* the hero of Mrs. Humphry Ward's latest theological novel, stands out before the reader. And the environment suits fitly the sombre figure of the recluse himself, upon whom the devout practice of his Faith in its austerest form has had, from the circumstances of his life, its slow but certain effect, till his character, naturally morbid and self-contained, has become as grey and shadow-full outwardly as the scene by which he is surrounded.

Descended from an old Catholic family—"Catholic" long before the Reformation—he keeps up the tradition and practice of faith by works of mercy. The maintenance of an orphanage and other good deeds has so impoverished the estate that everything connected with it has suffered in consequence. On all sides the lack of means is evident; in the worn and badly repaired roads; in the spacious stables surmounted by the Helbeck arms, now used as farm buildings; in gates hanging to ruinous posts; in the absence of servants within, and skilled labourers without, to keep up the dignity of the old place.

He is hurrying home, "his shoulders thrown back, a remarkably tall man, with dark head and short, grizzled beard. He held himself erect as a soldier holds himself; but he had never been a soldier." Later on he is described also as being a man whom his own people, women especially, always regarded with friendly, attentive eyes. On this evening, after an absence of fifteen years, he is expecting his widowed sister, Augustino Fountain, and her stepdaughter, Laura. Since her marriage to an agnostic Fellow of a Cambridge College (the son of a Lancashire yeoman and "a convinced democrat, a man who had reasoned right to his Radical opinions that common folk must do without") Helbeck had had little communication with his sister. Her union with Fountain had broken every family tradition. For the Helbecks had two distinguishing traits—pride of race and loyalty to the faith of their fathers. During the lifetime of Fountain Augustino had troubled little about the ordinances of religion. But the day after his death "she returned to her Catholic duties," and her present visit is the result of a reconciliation with her brother. Her stepdaughter was but a child when her father re-married. Now she is grown up and dominates her weak little stepmother, for whom she seems to have real affection, in the arbitrary, kindly way which her weak will, and fragile health, seems to necessitate.

Laura, as we first know her, is the reverse of attractive, in spite of her green eyes and gift for music. She is flippant, often vulgar; the yeoman blood comes out, in spite of a refinement of appearance. A born "heretic" also. But yet, as the story goes on, we are moved to sympathise with her, in spite of much that irritates, in her wilful defiance of other people's wishes and prejudices. Love for her father's memory is the moving, and at the same time disturbing, element in her life. All she had learnt from him was in direct opposition to everything by which, since her arrival at Bannisdale, she had been surrounded. The frequent services in the chapel attached to the house; the absence of any congenial society; the presence of Roman priests and dull Sisters of Mercy, all served to offend her. Her father's teaching had left her spiritual life void and empty; to her were available none of the solaces which the Church offers to its faithful children in the hour of suffering and bereavement. Sympathy she did not expect, whilst unconsciously craving for it; distraction she must have at all hazards. Poor little tortured soul! To whom could she turn? In the neighbourhood were relatives of her father. To them she would fly. Blood was thicker than water; they would understand and welcome her.

There are many episodes connected with her intimacy with these yeoman cousins which can only be explained by an inherited vanity. Her cousin Hubert whom she encourages in his boorish attentions, had but one claim to her forbearance, the tie of blood, and the common love of music which both possess; otherwise it is impossible to imagine, except from pure wilfulness or vanity, that anyone with the refinement we are constantly reminded as being natural to her, could have suffered for a moment the attentions of folks so unspeakably ignorant and vulgar. But these attentions were not only suffered, but on more than one occasion sought, by Laura herself. However, it is not round the Mason episodes that the interest of the story centres. These may pass; it is with Helbeck and Laura that we have to do. We must not forestall the interest which gathers round them as the story grows, but leave it to our readers to follow their fortunes to their tragic end, for themselves. Some may perhaps look upon Helbeck as a "bigot" or "martyr," as their inclination dictates; but we think that too much faith is better than no faith, when one contrasts the effect that the practice of the former has upon the life of our hero, and the loss of self-control occasioned by the want of it, in an undisciplined, self-directed life; with all its restless, reckless, self-seeking as evinced in the character of Laura. And, again, we are touched in speaking of her. Poor little heroine! With her artistic, dramatic temperament, torn by passionate devotion to her lover, and attracted, yet repelled, by the faith which he professes; drifting on the ocean of spiritual unrest; longing, yet fearing, to enter the Church of which he is so devout a member: she at length puts an end to all doubts and difficulties herself. "Helbeck of Bannisdale" may not diminish from a literary reputation already made, but it will not add to it, we think. It is unsatisfactory, as are so many of Mrs. Ward's books. It leaves one with a sense of something wanting. This may be due to sense of unrest, of something striven after, but not reached, by the writer. But, however that may be, there is no lack of interest in the story. Even when its methods are most disappointing, it is always interesting. The minor characters, if not attractive personally, are well filled in; and Leadham, the priest, a recent Oxford 'vert, is the most suggestively interesting of them all.

*Helbeck of Bannisdale. By Mrs. Humphry Ward. (London: Smith, Elder, and Co., 15, Waterloo Place. 6s.)

### The Book World for Women and Nurses.

[We invite Correspondence, Criticism, Enquiries, and Notes on Books likely to interest Women and Nurses. Address, Editor, THE HOSPITAL (Nurses' Book World), 28 & 29, Southampton Street, Strand, London, W.C.]

Silver Thorns. Florence H. Davies. (London: Saxon and Co. 1898. Price 1s. 6d.)

"Silver Thorns," by Florence H. Davies, contains six short stories illustrative of certain pathetic aspects of life, principally centred in the East-end. Miss Davies shows some decided artistic feeling, and a marked sense of sympathy with suffering humanity. "John, the Newsboy," which is the first st[illegible]y in her book, is, perhaps, the strongest example of thi[illegible]nd John's simple tragic little history will appeal to its r[illegible]s. "Silver Thorns" is prettily bound in a manner whic[illegible]edounds to the credit of the publishers, and the paper and printing are both most excellent.

### Our American Letter.

THE Johns Hopkin Training School for Nurses won the first prize for typhoid fever charts at the recent nurses' exhibit at the International Health Exposition at New York.

Miss Gertrude W. Moore, who has held the responsible position of superintendent of the Lady Stanley Institute for Trained Nurses, Ottawa, Canada, for the last seven years, has resigned. This post involves the superintendenceship of four institutions, namely, the County Carleton General Hospital, the Hospital for Contagious Diseases, and the Ottawa Maternity Hospital, as well as the Lady Stanley Institute. Miss Moore has won the cordial appreciation of her fellow workers, which was expressed by a parting gift of a handsome gold watch.

Miss Diana C. Kimber's vacant post as superintendent of the Training School for Nurses, Blackwell's Island, has been filled by Miss Mary S. Gilmore.

An interesting letter from Mrs Bonham-Carter has been received by Miss Wadley in response to a request that Miss Nightingale would allow her name to be used as a patroness of the Nurses' Educational Exhibit. Miss Nightingale refused, as it is her invariable rule never to lend her name as patroness or in any other connection to a public institution or undertaking in which she is unable to take an active part or exercise real control, and this she is often precluded from doing in consequence of her ill-health. Miss Nightingale takes much interest in the many successful workers in the United States for the development of sound nursing principles and nurse-training.

Three prizes were bestowed on the graduates of the New Jersey Training School for Nurses at the eighth annual commencement meeting. The "President's Prize," a gold cross, was given to the one who passed the best general examination. The writer of the best essay on a subject appertaining to nursing received the gold medal, which is "Faculty Prize," and a second gold medal was awarded by the Board of Managers to the graduate presenting the best report of the surgical clinics held at the Cooper Hospital.

### Everybody's Opinion.

[Correspondence on all subjects is invited, but we cannot in any way be responsible for the opinions expressed by our correspondents. No communication can be entertained if the name and address of the correspondent is not given, as a guarantee of good faith but not necessarily for publication, or unless one side of the paper only is written on.]

### HOSPITAL CONVALESCENT FUND.

"E. M. W." writes: Your letter I received on Saturday night, containing the postal order, for which I thank you. I have been favoured with splendid weather hitherto, and feel much better. Miss S. is very kind and considerate, and I am very happy here.

"NURSE S." writes: I think St. Leonards a delightful place, and I feel much better although I am not able to walk much. G—— (the home) is comfortable, and Miss G—— (the principal) has kindly given me a small bedroom, it being vacant at present; but I may have to give it up should it be required before the end of my stay. I hope to be able to remain in it. A separate room is a great boon to me, as since my illness I have not slept well. I trust you will accept my thanks for the trouble you have taken in procuring for me this delightful change, which cannot fail to benefit my health. I hope some day to return the cost to the Convalescent Fund.

### MATRONS' COUNCIL MEETING.

"LAURA D." writes: I have been very much interested in the letter of a "Constant Reader" in July 2nd's issue, and can heartily endorse all she says with regard to the practical training received in small country hospitals. It seems to be very unfair and unjust to speak slightingly of their capabilities for training, which, as a rule, are far greater than the large so-called "training schools." I speak entirely from personal experience, as I began my nursing career in a small country hospital of seventy beds. There were three of us probationers, and the head nurse was a practical woman, experienced in every branch of nursing, including midwifery. We had no resident doctor, and in consequence had to depend upon ourselves a great deal, for it was an understood thing that our hard-worked surgeon should only be summoned in cases of emergency. There were generally plenty of dressings, and these we always did ourselves, and many other things besides, which the nurses from the large "training schools" never seem to think of. At the end of two years I obtained a post in a large hospital, where very speedily I found a difference. It is very nice, no doubt, to telephone for the house surgeon a dozen or so times a day, but for my part I used to feel quite ashamed to see a doctor fetched into a ward on the most trivial excuse. Since then I have met nurses of every variety of training, and have found invariably that those from some small, obscure country hospital or infirmary are by far the best for a practical knowledge of things. It sounds very clever, no doubt, to talk learnedly of "reflexes" and the "advantages of the anti-toxin treatment," but give me the one who never loses her head in an emergency, who can prepare an accident case swiftly and carefully for the doctor, or know exactly what to do in the case of a fit. These are the sort of nurses who can be trusted with serious cases, which many from the large hospitals have never even helped to nurse. It is sometimes said that now that everybody flocks to London for training the country nurse will stand no chance of getting good appointments; but, having been very lucky myself, I can only hope for equal success for those of my sisters who are fortunate enough to get their training in the country.

### Minor Appointments.

LINCOLN COUNTY HOSPITAL.—Miss Mary G. Wilkinson has been appointed Matron's Assistant at this hospital. She was trained and certificated at Nottingham General Hospital, and has held the post of charge nurse both in the children's and latterly in the female medical and surgical wards of that institution. She is a registered member of the Royal British Nurses' Association.

CHESTERFIELD UNION WORKHOUSE INFIRMARY.—On July 9th Miss Isabella Rowe was appointed Superintendent Nurse of this infirmary. She was trained in Dublin Orthopædic Hospital for two years, and in the Workhouse Hospital, Liverpool, for nine months, and has been sister at St. George's-in-the-East Union Infirmary for four years.

WHITCHURCH COTTAGE HOSPITAL.—In November last Miss Lavinia Wilson was appointed Assistant Nurse at this hospital. She was trained at the South-Eastern Hospital, London, and has held appointments at the City Hospital, Liverpool, the City Hospital, Sheffield, and at the Hednesford Accident Home.

CHELSEA HOSPITAL FOR WOMEN, FULHAM ROAD.—Miss Margaret Oakley was appointed Charge Nurse at this hospital on July 14th, 1898. She was trained at Grimsby District Hospital, and was afterwards ward and theatre sister at Worcester General Hospital.

PINNER.—Miss Margaret J. Phelp has been appointed Parish Nurse at Pinner. She was trained at Fir Vale Infirmary, Sheffield, and holds the L.O.S. certificate. She has lately been on the staff of the Blackheath and Richmond Private Nursing Institution.

ISOLATION HOSPITAL, NOTTINGHAM.—Miss Hilda M. Sykes has recently been appointed Sister of this institution. She was trained at the Royal Infirmary, Truro, and at the Fever Hospital, Taunton, and has been theatre nurse at her first training school.

NATIONAL ORTHOPÆDIC HOSPITAL.—Miss Lily de Chastelain was appointed sister to this hospital on July 2nd. She was trained at St. Saviour's Infirmary, Dulwich, and has been four months staff nurse at the New Hospital for Women, Euston Road.

### For Reading to the Sick.

### CHRISTIAN SERVICE.

### Verses.

Teach me my God and King,
In all things Thee to see,
And what I do in anything
To do it as to Thee.

A servant with this clause
Makes drudgery divine;
Who sweeps a room as for Thy laws
Makes that and th' action fine.
—*Herbert.*

What is left for us save in growth
Of soul to rise
From the gift looking to the Giver,
And from the cistern to the river,
And from the finite to infinity,
And from man's dust to God's divinity?
—*Browning.*

Up! God has formed thee with a wiser view;
Not to be led in chains, but to subdue!
Calls thee to cope with enemies, and first
Points out a conflict with thyself—the worst!
—*Cowper.*

Arise! for the day is passing,
And you lie dreaming on!
The others have buckled their armour,
And forth to the fight are gone.
A place in the ranks awaits you,
Each man has some part to play;
The past and the future are nothing
In the face of the stern to-day.
—*A. Procter.*

Whene'er a noble deed is wrought,
Whene'er is spoken a noble thought,
Our hearts in glad surprise
To higher levels rise.

The tidal wave of deeper souls
Into our inmost being rolls,
And lifts us unawares
Out of all meaner cares.

Honour to those whose words or deeds
Thus help us in our daily needs,
And by their overflow
Raise us from what is low.
—*Longfellow.*

### Reading.

Christian service in its full idea is a phrase practically co-extensive with Christian life; and Christian life is, in the intention of the Gospel—nothing less, nothing narrower, than the whole life of the Christian; morning, noon, and night; alone, in private, in society, in public, at all times and in all places. From one point of view, and that a most important point, he not only is a servant of the Lord, but he is a servant of the Lord in such a way, under such conditions, that the whole action of his life falls under the description of his service. As he always exists, as a Christian, in and by his Master, so he always exists for his Master. He has, in the reality of the matter, no dissociated and independent interest. Not only in preaching and teaching and bearing articulate witness to Jesus Christ does he, if this life is true to its idea and its secret, "live not unto himself"; not with aims which terminate for one moment in his own credit, for example, or his own comfort. Equally in the engagements of domestic life, of business life, of public affairs; equally (to look towards the humbler walks of duty) in the day's work of the Christian servant, or peasant, or artizan; "whether he lives, he lives unto the Master, or whether he dies, he dies unto the Master"; whether he wakes or sleeps, whether he toils or rests, whether it be the term or the vacation of life, "whether he eats or drinks, or whatever he does," he is the Master's property for the Master's use.—*H. C. G. Moule.*

### Notes and Queries.

**The contents of the Editor's Letter-box have now reached such unwieldy proportions that it has become necessary to establish a hard and fast rule regarding Answers to Correspondents. In future, all questions requiring replies will continue to be answered in this column without any fee. If an answer is required by letter, a fee of half-a-crown must be enclosed with the note containing the enquiry. We are always pleased to help our numerous correspondents to the fullest extent, and we can trust them to sympathise in the overwhelming amount of writing which makes the new rules a necessity.**

**Every communication must be accompanied by the writer's name and address, otherwise it will receive no attention.**

*Completion of Training.*

(155) Please can you tell me which hospital I might get general training? I went to one of the hospitals last week, but was refused on the "grounds of having been in another institution." I cannot afford to give my services.—*E. L., Fulham.*

Write to the Matron, the City of London Hospital for Diseases of the Chest, Victoria Park, E. She is arranging a system which will afford nurses with some training an opportunity of completing it and earning a three-year certificate. Probationers receive £10 for the first year, and £16 for the second. It is very difficult to obtain completion of training.

*L.O.S.*

(156) I want to train, privately if possible, for the L.O.S. certificate. Can you tell me anything about the training and reported success of the pupils of Fram Gotla, who advertises in your columns?—*L. M. L.*

You must apply for references from Fram Gotla in the usual way. Though great care is taken that only reliable advertisements appear in our columns, it is impossible for us to certify that the details will fulfil your requirements.

*Mildmay Hospital.*

(157) Can you recommend Mildmay Hospital, Bethnal Green, E., as a training school? My friend, for whom I write, does not wish to become a missionary, but a well-trained nurse. Her health broke down when training in a very large provincial hospital, and she wishes to enter a smaller one, where the work is not quite so hard.—*Red Tape.*

Under such circumstances as to health, we should strongly recommend your friend not to come to London. The Mildmay Hospital is a recognised training school, and the three-year certificate qualifies its holder for important offices.

*A Disqualification.*

(158) I have lost an eye in consequence of an accident. Will this disqualify me for the nursing profession? The sight of the other eye is perfect.—*K.*

No one can answer such a question but a medical man who has examined your eyes. It is quite possible, if you are suitable for the work in other respects, that you may be accepted as a probationer.

*Weir-Mitchell Treatment.*

(159) Will you kindly enlighten me as to what Weir-Mitchell treatment is?—*Inquirer.*

The Weir-Mitchell treatment was introduced by a doctor of that name. By it obstinate nervous diseases are treated by special diet, massage, isolation, and prolonged rest.

*Rheumatism.*

(160) Do you know of any home to which a patient suffering from chronic rheumatism could be sent for the winter, or as a permanency. She is 39, a domestic servant, and almost helpless. She has been in her present situation sixteen years, but her mistress is unable to pension her. I have tried to borrow Burdett's Annual, but have not been successful.—"*Fellow Feeling.*"

There is a ward for women at St. Lucy's Home for Incurables, Leamington. The Midland Counties Home for Chronic and Incurable Diseases, Leamington, admit some patients free. There is also the Thompson Memorial Institution for Incurables, Lisburn. Will our readers kindly help "Fellow Feeling" if they can?

*Cheap Monthly Training.*

(161) Kindly tell me where I could get a certificate as a monthly nurse with the least expense. I could not afford Queen Charlotte's.—*No. 281.*

The East-End Mothers' Home, 396, Commercial Road, E., gives the cheapest training. It is excellent.

*Dispensing for Ladies.*

(162) I should be glad if you would give me your opinion as regards dispensing for ladies. I am anxious to learn, but fear failure to obtain an appointment after spending the money in qualifying for it. What salaries do dispensers get?—*Lal.*

There is certainly a good opening for lady dispensers. The salaries range from £80 to £120.

*How to Become a Nurse.*

(163) I am anxious to become a hospital nurse. Can you tell me where to apply? I prefer a London school. I am 34 years of age.—*P. A.*

You should have sent your name as well as address. Apply direct to the Matron at one of the London hospitals. State your age. You have nearly reached the limit. See Honnor Morten's "How to Become a Nurse" (2s. 6d., from the Scientific Press, London).

(164) Kindly tell me where I could train as a nurse. I should prefer Scotland. I am nineteen and a half.—*F. A. K. C.*

You are too young for many hospitals. The Dumfries Royal Infirmary or the Aberdeen Hospital for Incurables might accept you.

